# Survival analysis of laryngeal squamous cell cancer, considering different treatment modalities and other factors influencing survival – a monocentric retrospective investigation

**DOI:** 10.1007/s00405-025-09229-8

**Published:** 2025-02-17

**Authors:** Gábor Dénes Répássy, András Molnár, Stefani Maihoub, Dóra Hargas, László Tamás

**Affiliations:** 1 Department of Otorhinolaryngology and Head and Neck Surgery, Semmelweis University, Szigony u. 36, Budapest, H-1083 Hungary; 2 Department of Voice, Speech and Swallowing Therapy, Semmelweis University, Vas u. 17, Budapest, H-1088 Hungary

**Keywords:** Larynx cancer, Survival, p16 expression, Surgery, Chemoradiation, Influencing factors

## Abstract

**Purpose:**

This study aimed to investigate the factors affecting laryngeal cancer survival.

**Methods:**

This study retrospectively analysed laryngeal cancer types, treatment options, and potential factors influencing survival.

**Results:**

77 patients (26.27%) had supraglottic laryngeal cancer, 209 (70.13%) had glottic laryngeal cancer, and 7 (3.6%) had subglottic laryngeal cancer. Common comorbidities such as type 2 diabetes mellitus, chronic obstructive pulmonary disease, and coronary disease were observed in 13.65%, 11.9%, and 22.18% of the patients, respectively. Smoking was detected in 88.05% of the patients, while 56.3% reported regular alcohol consumption. The study found that hemilaryngectomy and supraglottic horizontal resection led to significantly longer survival compared to other treatment options (i.e., total laryngectomy, supracricoid horizontal partial laryngectomy, transoral laser cordectomy, chemoradiation, chemotherapy, and radiotherapy), *p* = 0.000*. Glottic cancers tend to have longer survival when considering laryngeal cancer locations; however, this difference was statistically insignificant (*p* = 0.640). Statistical comparisons showed significantly longer survival rates for surgical treatments in stages 1 (*p* = 0.007*) and 4 (*p* = 0.007*). Factors such as coronary artery disease, higher ECOG performance status, advanced ‘*N’* stages, and higher tumour grades were found to significantly worsen survival, as determined by a Cox proportional hazards model.

**Conclusion:**

The study revealed that factors such as coronary disease, patients’ functionality, ‘*N’* stages, and tumour grade significantly impacted survival rates. Furthermore, the study found that supraglottic horizontal resection and hemilaryngectomy resulted in the longest survival. Surgical methods were associated with significantly longer survival rates in disease stages 1 and 4.

## Introduction

Laryngeal cancer can spread to the supraglottic, glottic, and subglottic regions. It is the second most common type of head and neck cancer, with an estimated 184,615 new cases reported in 2020. Central and Eastern Europe have the highest mortality rates associated with this type of cancer [1]. Squamous cell carcinoma is the most common histological subtype, and almost all squamous cell variants are found in the laryngeal region [2, 3]. Generally, laryngeal cancer shows a higher occurrence in males, with smoking and regular alcohol consumption being the primary risk factors [4]. Additionally, comorbidities such as type 2 diabetes mellitus (T2DM) [5], chronic obstructive pulmonary disease (COPD) [6] and coronary disease [7] can also impact general health status and survival. However, p16-related oncogenic pathways have been suspected and described in laryngeal cancer, but the effects of p16-positive and HPV-related oncogenicity remain unclear in laryngeal cancers [8].

The treatment of laryngeal cancers can be challenging, often necessitating multimodality treatment for advanced cases. In cases of early laryngeal cancer, patients may be offered surgery or primary radiotherapy as treatment options. For this, transoral laser surgery or open partial laryngectomy can also be choice. Surgical treatment may include various modalities, with an emphasis on options that lead to better functionality and rehabilitation. Therefore, transoral laser surgery or open partial laryngectomy could be viable choices [9, 10]. In cases of advanced laryngeal cancer (T3-4N0-3 disease), multimodality treatment is often necessary, and partial surgeries may not be feasible in some instances. Multimodality treatment refers to either surgery followed by radiotherapy or chemoradiation. In contrast to early-stage diseases, the treatment of advanced laryngeal cancers involves level I evidence, achieving good locoregional control using chemoradiation, which enables larynx preservation. For T3 diseases, chemoradiation should be considered, and in cases of tumour recurrence, salvage total laryngectomy following chemoradiation should be indicated. In T4a cases, total laryngectomy with adjuvant radiation yields similar results for locoregional control as chemoradiation or salvage surgery but with better survival rates. For T4a cases, it is not recommended to use chemoradiation, as it leads to lower survival rates [10, 11]. In some T3 and T4a cases, partial surgeries with an external approach can be beneficial, providing better functionality and similar survival rates compared to radical surgeries [12]. After salvage surgeries, a higher rate of complications, such as pharyngocutaneous fistulae and slow wound healing, may be expected [13, 14].

Surgeries for laryngeal cancers can include total laryngectomy and partial laryngeal surgeries, such as transoral cordectomy, hemilaryngectomy, supraglottic horizontal resection and supracricoid horizontal partial laryngectomy with the most important ones among them mentioned. Total laryngectomy is the complete removal of the larynx, requiring ventilation through a tracheostomy tube due to airway separation. This procedure is primarily carried out for advanced T3 and T4 cancers. Transoral cordectomy, typically performed for T1 cancers of one vocal cord, involves the resection or removal of the vocal cords, often utilising laser or robotic assistance. Type IV cordectomy primarily involves the complete removal of the vocal cord, including the epithelia, ligaments, and muscles, from the anterior to the posterior commissure. Type I refers to subepithelial resection, while type II is sub-ligamental resection. Type III involves a transmuscular resection with the potential removal of the ventricular fold. For T1 and T2 supraglottic cancers, supraglottic horizontal resections or Alonso surgery involve removing the entire laryngeal vestibule, with the incision line running horizontally through the laryngeal ventricle of Morgagni. This procedure eliminates all supraglottic structures and the pre-epiglottic area. Hemilaryngectomy refers to a modified surgical procedure of Hautant. It involves a vertical skin incision and the removal of the tumorous hemilarynx, including the vocal cord, anterior commissure, ipsilateral arytenoid cartilage, and most of the ipsilateral thyroid cartilage. Nowadays, the cricoid cartilage is usually spared in this procedure. When performing a supracricoid horizontal partial laryngectomy, the thyroid cartilage, vocal cords, arytenoid regions, epiglottis (i.e., cricohyoidopexy) and the parapharyngeal space are completely removed, while preserving the hyoid bone and the cricoid cartilage. This procedure benefits glottic and glotto-supraglottic cancers, with some selected cases of T4 supraglottic and glottic cancers [15, 16].

In addition to enhancing surgical treatments for laryngeal cancers, there have been advancements in non-surgical treatment options as well. When considering radiotherapy, a total dose of 66–70 Gy is typically applied in 33–35 fractions (2 Gy per fraction). Postoperatively, radiation dosages of 54 to 60 Gy are recommended for locally advanced tumours, 60 to 66 Gy for complete surgical resections, and 60 to 66 Gy for R1 resections. A dosage of 50 to 66 Gy is used for the lymph node areas depending on extracapsular spreading. It is important to initiate radiotherapy as soon as possible, preferably within two months after surgery. For instance, a previous study demonstrated the positive impact of postoperative adjuvant radiation on pT4aN0 glottic cancers [17]. Chemotherapy involves a combination of cisplatin and 5-fluorouracil using body-surface area-based dosing, specifically 100mg/m^2^ intravenously every three weeks [18]. However, since 2008, the ‘EXTREME’ protocol has been introduced, which combines cetuximab, an epidermal growth factor receptor monoclonal antibody, with chemotherapy options such as cisplatin, carboplatin, or 5-fluorouracil. Previous trials indicated that this treatment combination improved overall survival (OS) in patients with recurrent or metastatic squamous cell head and neck cancers as a first-line option [19]. Furthermore, since 2019 the pembrolizumab, a PD-L1 receptor inhibitor humanised antibody and chemotherapy scheme is applied. Pembrolizumab monotherapy and its combination with platinum and 5-fluorouracil have significantly improved OS in patients with recurrent or metastatic squamous cell head and neck cancers, based on previous trials [20]. Adjuvant chemoradiation is recommended for high-risk cases with histological evidence of regional lymph node metastases, extracapsular extension of the nodal disease, microscopically involved tumour margins, and in some cases, when the resection margin is less than 2 mm. In some cases, docetaxel induction chemotherapy may also be applied. Eligibility for chemotherapy is assessed using the ECOG (Eastern Cooperative Oncology Group) scale, and routine laboratory testing, including white blood cell and platelet counts and creatinine clearance, is performed before chemotherapy. According to some previous research reports, it is possible to perform partial surgeries even after chemoradiation [11].

Despite numerous changes and improvements in the management of laryngeal cancer, the 5-year survival rates have not increased as anticipated. 5-year survival depends on disease stage and location. For example, a previous investigation found 5-year disease-specific survival (DSS) of 100% for T1a, 95% for T1b, 78% for T2, 79% for T3, and 53% for T4 glottic cancers. In cases of supraglottic cancers, the observed DSS values were 68%, 54%, 72%, and 59%, respectively [21]. Over the past 150 years, there has been a significant shift in the treatment of laryngeal cancers. Previously, radical surgeries were the norm, even for early-stage disease [22]. However, in the current era of treating laryngeal cancers, the focus is on partial surgeries with organ preservation, aiming for improved functionality and similar survival rates compared to radical surgeries [23]. In addition to external methods, significant progress was made by utilising CO2 laser surgeries in the early stages of laryngeal cancer [24]. Since then, investigations have found similar survival rates in T1 and T2 laryngeal cancers when comparing surgeries and primary radiotherapy [25]. As a part of the advancement in treatment options, multimodality treatments have significantly improved outcomes in laryngeal cancers. Better survival rates have been observed with chemoradiation compared to radiotherapy [26]. However, this observation is relevant only to advanced stages of laryngeal cancers [27].

Despite notable advancements in treating laryngeal cancers, survival rates have remained largely unchanged. To address this issue, the primary objective of this study was to analyse the various treatment approaches and potential factors influencing survival in laryngeal cancers.

## Materials and methods

### Study population and design

A total of 293 patients diagnosed with squamous cell larynx cancer diagnosed between July 2002 and March 2023 were enrolled in this investigation. Each patient was diagnosed and treated in the Department of Otorhinolaryngology and Head and Neck Surgery of Semmelweis University with at least one year of follow-up. The diagnosis and treatment followed the latest National Comprehensive Cancer Network^®^ (NCCN) guideline for Head and Neck Cancers [28]. Inclusion criteria included the diagnosis of squamous cell larynx cancer in any laryngeal region by an otorhinolaryngologist and histological examination, patient consent to participate in this investigation, eligible clinical data, and at least one year of follow-up. Those patients with other types of head and neck cancer, other primary tumour, lacking clinical data, lost in follow-up, or not consenting to participate, were excluded from this study. Clinical data, including clinical examinations, treatment, survival factors (e.g., smoking, alcohol consumption, TNM, stage or p16 expression), and comorbidities (e.g., T2DM, COPD, and coronary disease), were obtained from the University’s electronic medical system. For the purpose of weight loss, a duration of 6 months was established as the timeframe. The treatment modalities were categorised as total laryngectomy, supracricoid horizontal partial laryngectomy, supraglottic horizontal resection, hemilaryngectomy, transoral laser cordectomy, chemoradiation, chemotherapy, radiotherapy, including palliative irradiation, and patients who did not consent to or could not receive treatment due to poor health were categorised as not having received treatment. Larynx cancer was categorised based on its location as supraglottic, glottic or subglottic cancer.

### Clinical examinations, diagnostic work-up

All patients were diagnosed with laryngeal cancer by a specialist, using a general otorhinolaryngological examination and laryngoendoscopy. After the diagnosis, staging examinations were offered, including CT scans of the neck, chest, and abdomen using contrast. Alternatively, PET-CT scans were recommended in combination with contrast-enhanced neck CT scans. Laryngomicroscopy under general anaesthesia was performed to obtain cancer tissue for histological analysis and to observe local tumour spreading. Additionally, p16 immunohistochemistry was applied during the histological examinations, as detailed below. Staging classification for laryngeal cancers was determined using the most recent American Joint Committee on Cancer (AJCC) staging manual [29]. The study was conducted according to the guidelines of the Declaration of Helsinki and approved by the Institutional Ethics Committee of Semmelweis University (protocol code: SE IKEB 105/2014; approval date: 29 May 2014).

### Comorbidities, potential influencing factors

Analysis was conducted on the co-occurrences of T2DM, COPD, and coronary artery disease and their potential impact on survival, considering the general characteristics of the populations. As a main risk factor for laryngeal cancers, smoking and regular alcohol consumption was also considered. As it might influence survival, body mass index (BMI) and the potential impact of weight loss on survival were also investigated. Patients’ daily functionality was evaluated using the ECOG scale, and its score was included as a potential influencing factor. TNM stages and grades were also taken into account.

### p16 immunohistochemistry

For the p16 immunohistochemistry, 4 μm histology slides were stained using a Benchmark Ultra Plus automated system (Roche, Basel, Switzerland). The process began with deparaffinisation of the histology slides in EZ Prep Solution (Roche, Basel, Switzerland). Subsequently, a cell conditioning solution (pH = 9) for heat-induced epitope retrieval was applied (Roche, Basel, Switzerland) at a temperature of 95 °C for 30 min. Following this, a drop of UV INHIBITOR (Roche, Basel, Switzerland) was used to inhibit endogenous peroxidase activity and left to incubate at 37 °C for 6 min. A p16-INK4 monoclonal antibody (Cell Marque, Rocklin, CA) was then incubated at 37 °C for one hour with a dilution of 1:100. The binding of the primary antibodies was visualised using the OptiView Amplification kit (Roche, Basel, Switzerland). Nuclear counterstaining was achieved using Haematoxylin II (Roche, Basel, Switzerland). Additionally, a diluted Reaction Buffer Concentrate (Roche, Basel, Switzerland) was used for washing. A positive p16 staining was classified as a distinct cytoplasmic and nucleolar positive reaction in at least 70% of the tumour tissue [30].

### Statistical analysis

Data processing was performed using IBM SPSS V25 software (IBM Corporation, Armonk, NY, USA). Data normality was checked using the Shapiro**–**Wilk test. Continuous variables were provided as mean ± SD or median values, based on the normality of the data. The Mann**–**Whitney *U* and Kruskal**–**Wallis tests were used for analysing significant differences between each group. To analyse survival, survivorship curves (Kaplan**–**Meier) were plotted, and the influence of different factors on survival was analysed using the log-rank (Mantel**–**Cox) test. Moreover, to analyse the effects of multiple variables on survival, the Cox (Proportional Hazards) regression was applied. This model included the following parameters: therapy, age, sex, p16 expression, BMI, weight loss, T2DM, COPD, coronary artery disease, smoking, and regular alcohol consumption, TNM, and tumour grade. The Spearman’s correlation test was used to analyse the correlations between the parameters. A *p*-value under 0.05 was consistently considered statistically significant.

## Results

The basic clinical data of the examined groups is provided in Table 1.

**Table 1:** Basic clinical data of the study population

Category	
Age (years, mean ± SD)	64.02 ± 7.28
Sex (men/women)	247/46
Follow-up time (months, mean ± SD)	45.39 ± 38.94
**Location**, *n* (%)	
supraglottic glottic subglottic	77 (26.27%) 209 (71.3%) 7 (2.43%)
**P16 expression** (positive/negative)	
supraglottic glottic subglottic	30/47 33/176 0/7
**Therapy**, *n* (%)	
Total laryngectomy Supracricoid horizontal partial laryngectomy Supraglottic horizontal resection Hemilaryngectomy Transoral laser cordectomy Chemoradiation Chemotherapy Radiotherapy No treatment	81 (27.6%) 10 (3.4%) 57 (19.45%) 19 (6.48%) 12 (4.1%) 8 (2.7%) 49 (16.72%) 7 (2.38%) 50 (17.06%)
**Comorbidities**, *n* (%)	
T2DM COPD Coronary artery disease	40 (13.65%) 35 (11.9%) 67 (22.86%)
**Smoking**, *n* (%)	258 (88.05%)
**Regular alcohol consumption**, *n* (%)	165 (56.3%)

COPD = chronic obstructive pulmonary disease; SD = standard deviation; T2DM = type 2 diabetes mellitus

According to the table, the average age of patients with larynx cancer was approximately 64 years, indicating that older individuals are generally affected. Additionally, there was a significant male predominance in this study population. When comparing survival times by sex, no significant differences were found (*p* = 0.198, *Z*-score: 1.287, Mann*–*Whitney *U* test). When analysing the correlation between age and survival, no significant correlation was observed (rho = 0.089, *p* = 0.135) according to Spearman’s correlation test. The average follow-up time for this population was 45 months (3.75 years), with the longest follow-up period being 20.83 years. Most patients had glottic cancer (71.3%), followed by supraglottic (26.27%) and subglottic (2.43%) types. P16-positivity was observed in 21.5% of the entire study population. When considering cancer location, 38.9% of supraglottic, 15.8% of glottic, and 0% of subglottic cases presented p16-positivity. Regarding treatment modalities, the most common categories were total laryngectomy (27.6%), supraglottic horizontal resection (19.45%), and chemotherapy (16.72%). Approximately 17% of patients did not receive treatment. In terms of comorbidities, 22.86% had coronary artery disease, 13.65% had T2DM, and 11.9% had COPD, respectively. Smoking was reported in 88% of cases, and regular alcohol consumption in 56.3%.

**Fig. 1:**
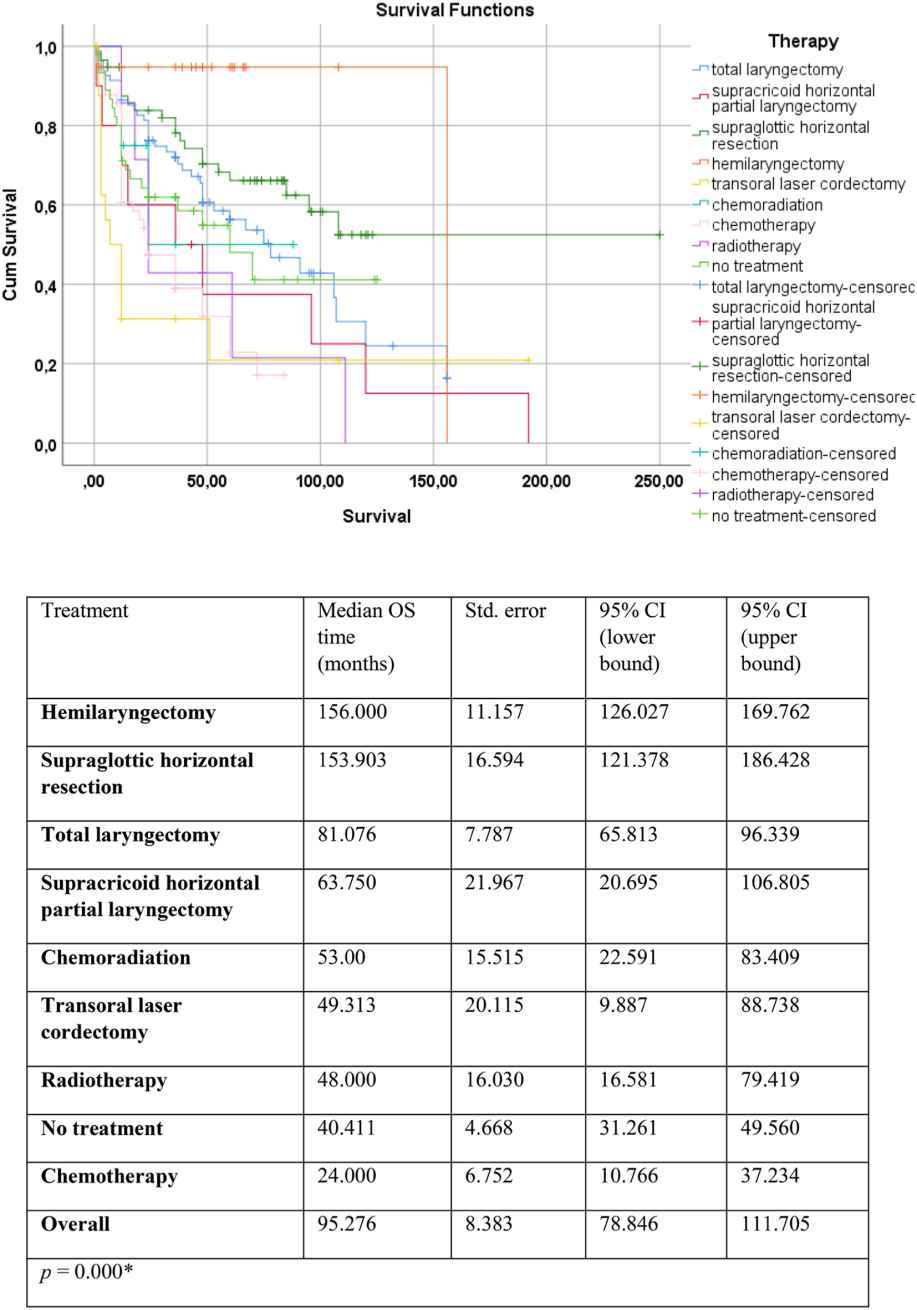
Kaplan–Meier analysis on survival (months) depending on different treatment modalities. The blue line represents total laryngectomy, the red line represents supracricoid horizontal partial laryngectomy, the dark green line represents supraglottic horizontal resection, the orange line represents hemilaryngectomy, the yellow line represents transoral laser cordectomy, the turquoise line represents chemoradiation, the pink line represents chemotherapy, the purple line represents radiotherapy, and the light green line refers to cases where no treatment was received. The *p*-value was calculated using the log-rank (Mantel–Cox) test (*p* < 0.05*). The asterisk (*) indicates a statistically significant difference. CI = confidence interval; OS = overall survival; Std. = standard OS = overall survival

In the next step of the investigation, the OS depending on treatment modalities were analysed. The results are depicted in Fig. 1.

Figure 1. reveals that patients who underwent hemilaryngectomy had the longest median survival of 156 months (95% CI: 126.027–169.762). A similar outcome was revealed for supraglottic horizontal resection with median OS of approximately 154 months (95% CI: 121.378–186.428). Conversely, the shortest median survival time of 24 months (95% CI: 10.766–37.234) was observed in the chemotherapy group. The log-rank test indicated a statistically significant difference in OS between the groups (*p* = 0.000*). Interestingly, a lower OS was achieved in the group that underwent transoral laser cordectomy than expected. This could be due to the fact that our patients who had transoral laser cordectomy were newly diagnosed and had relatively shorter follow-up times compared to the other groups (28.87 ± 19.95 months vs. 46.02 ± 31.17 months), with a statistically significant difference (*p* = 0.001*, *Z*-score: 3.26; Mann–Whitney *U* test).

**Fig. 2:**
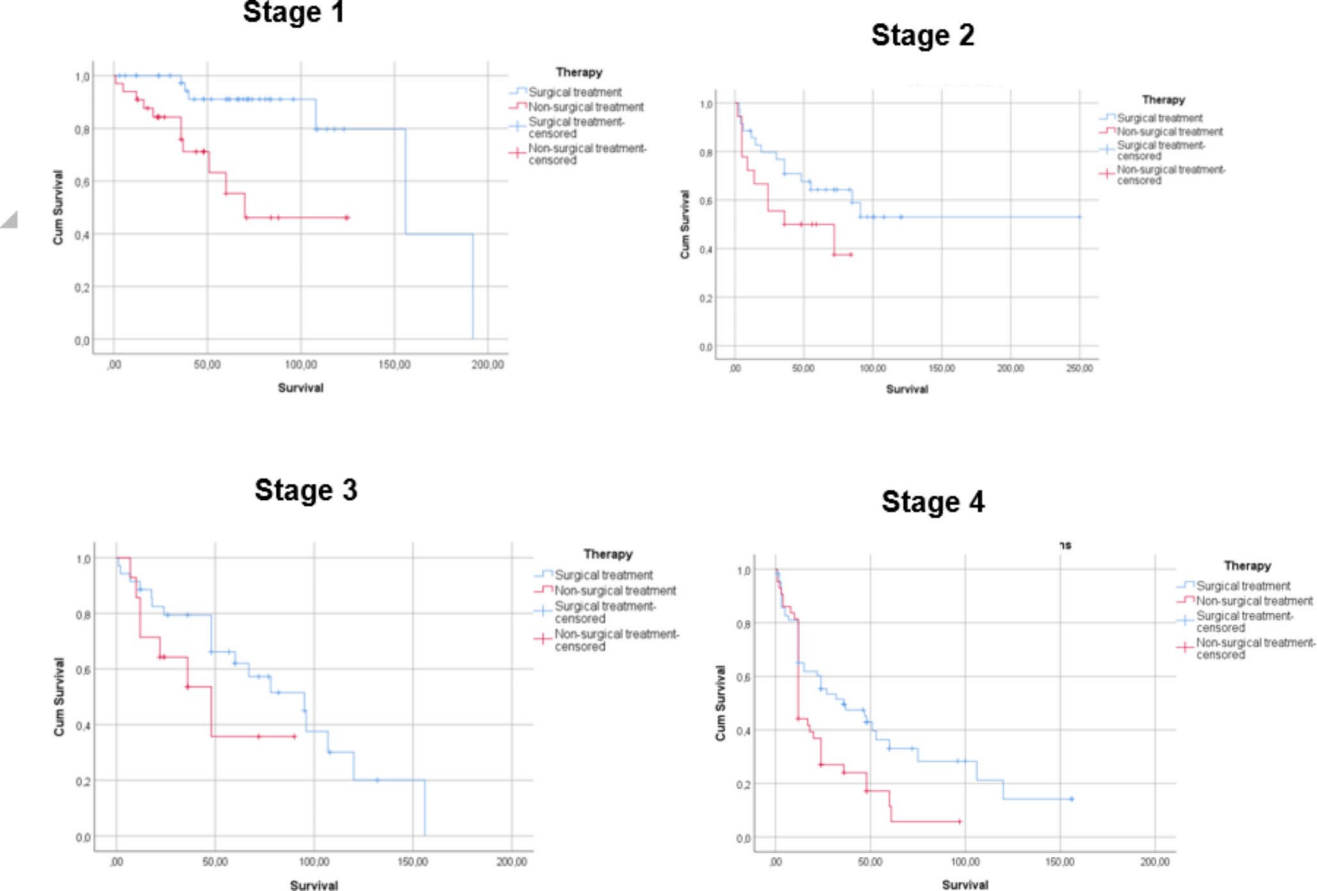
Kaplan–Meier analysis on survival (months) depending on larynx cancer stages and surgical and non-surgical treatment modalities. The blue line consistently indicates surgical treatment, while the red line represents non-surgical treatment. Non-surgical treatment modalities include primary radiotherapy for stage 1 and 2 groups, and chemoradiation for stage 3 and 4 groups

As the next step, a Kaplan–Meier analysis, depending on the larynx cancer stages and considering surgical and non-surgical treatment modalities, was performed, and the results are depicted in Fig. 2.

According to Fig. 2., patients with stage 1 laryngeal cancer showed significantly (*p* = 0.007*, log-rank test) longer survival rates in the surgical treatment group (median survival: 154.317 months; 95% CI: 124.566–184.068) compared to the non-surgical treatment group (median survival: 79.407 months; CI: 59.241–99.574). In stage 2 disease, the surgery treatment group showed a tendency for longer survival rates (median survival: 150.683 months; 95% CI: 110.918–190.448); however, this difference was not statistically significant (*p* = 0.112, log-rank test) when compared to non-surgical treatment options (median survival: 47.389 months; 95% CI: 31.246–63.531). A similar pattern was noted for stage 3 diseases, with no statistically significant differences (*p* = 0.145, log-rank test) between the surgical (median survival: 83.623 months; 95% CI: 63.494–103.752) and non-surgical treatment (median survival: 49.071 months; 95% CI: 28.869–69.274) groups. In cases of stage 4 laryngeal cancers, surgical treatment (median survival: 56.342; 95% CI: 40.253–72.430) has been associated with statistically significantly (*p* = 0.007*, log-rank test) longer survival rates compared to non-surgical treatment (median survival: 25.769; 95% CI: 17.674–33.865).

**Fig. 3:**
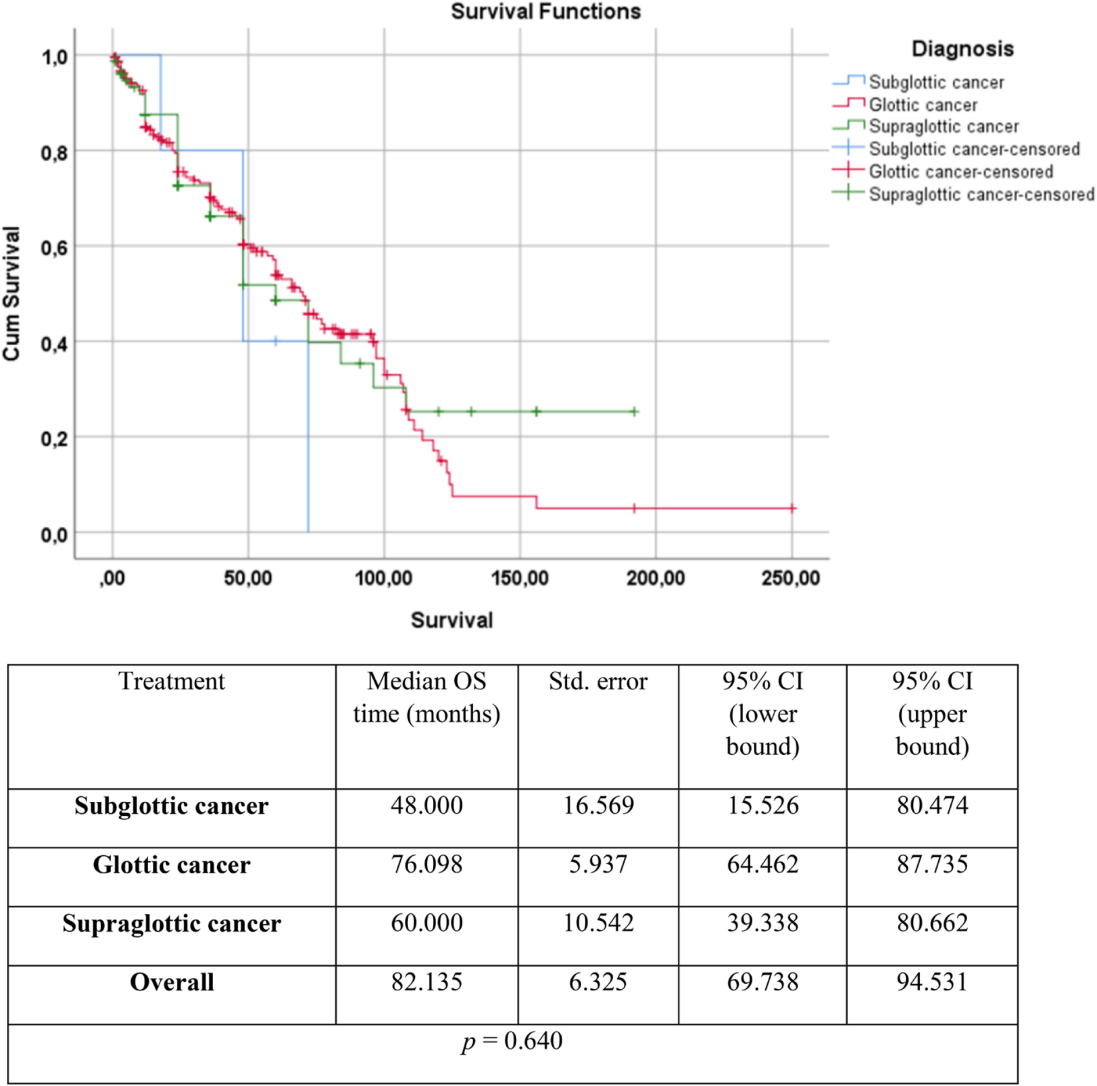
Kaplan–Meier analysis on survival (months) depending on different treatment modalities. The blue line represents subglottic cancer, the red line represents glottic cancer and the green line represents supraglottic cancer. The *p*-value was calculated using the log-rank (Mantel–Cox) test (*p* < 0.05*). OS = overall survival

To analyse the differences between the OS in supraglottic, glottic and subglottic cancers, an additional survivalship curve was plotted (Fig. 3.)

As depicted in Fig. 3., the Kaplan–Meier analysis revealed the longest median OS of 76.098 months (95% CI: 64.462–87.735) in glottic cancers, followed by slightly lower median OS of 60 months (95% CI: 39.338–80.662) in supraglottic glottic cancers. The lowest survival was found in subglottic cancers with a median of 48 months (95% CI: 15.526–80.474). However, the statistical analysis did not show a significant difference between the groups (*p* = 0.640), although there is a tendency for higher survival rates in glottic and supraglottic cancers.

In addition to comparing therapies on survival, the prediction of other factors on survival was also analysed, and the results are presented in Table 2.

**Table 2:** Cox proportional hazards model to analyse the effects of various factors predicting survival in larynx cancers

Factor	β	Std. Error	*p*-value	Exp. (β) (HR)
p16-positivity	-0.385	0.520	0.458	0.680
Smoking	0.677	0.489	0.166	1.968
Regular alcohol consumption	-0.163	0.262	0.534	0.850
T2DM	-0.134	0.406	0.741	0.874
COPD	0.476	0.361	0.188	1.610
Coronary artery disease	-0.054	0.275	0.039*	0.947
Sex	-0.200	0.231	0.386	0.819
Weight loss	0.114	0.354	0.748	1.120
BMI	0.044	0.155	0.775	1.045
ECOG	0.534	0.156	0.001*	1.705
’T’	0.171	0.115	0.139	1.186
’N’	0.508	0.226	0.025*	1.662
’M’	0.481	0.172	0.453	1.138
Grade	0.879	0.402	0.029*	2.409

BMI = body mass index; CI = confidence interval; COPD = chronic obstructive pulmonary disease; ECOG = Eastern Cooperative Oncology Group; HR = hazard ratio; Std. = standard; T2DM = type 2 diabetes mellitus; *T* = tumour, *N* = regional nodal metastasis, *M* = distant metastasis. Exp. (*β*) shows the hazard ratios. (*p* < 0.05*), the asterisk (*) indicates a statistically significant difference

In Table 2., it was found that smoking (*p* = 0.166; HR = 1.968), regular alcohol consumption (*p* = 0.534; HR = 0.850), and certain conditions like T2DM (*p* = 0.741; HR = 0.874) and COPD (*p* = 0.188; HR = 1.610) did not significantly predict survival in larynx cancers. BMI (*p* = 0.775; HR = 1.045) and weight loss (*p* = 0.748; HR = 1.120) also did not have a significant effect. P16-positivity (*p* = 0.458; HR = 0.680) and sex (*p* = 0.386; HR = 0.819) also did not significantly affect the results. However, coronary artery disease was identified as significant predictor of worse survival (*p* = 0.039*; HR: 0.947). Additionally, significantly better survival was associated with lower ECOG scores (*p* = 0.001*; HR = 1.705), ‘*N*’ stages (*p* = 0.025*; HR = 1.662) and lower tumour grades (*p* = 0.029*; HR = 2.409).

## Discussion

The study examined the survival rates of patients with laryngeal cancer based on different treatment methods and risk factors. Because laryngeal squamous cell cancer presents with diverse clinical characteristics and treatment options, survival outcomes can vary widely among patients. Our findings indicated that coronary artery disease, higher ECOG performance status, advanced tumour stages, primary chemotherapy, and radiotherapy were all linked to lower OS rates. When comparing survival rates for larynx cancer across different stages and treatment options, significantly longer survival rates were found for patients who underwent surgical treatments in stages 1 and 4. For stages 2 and 3, although patients who received surgical treatment also tended to have longer survival rates, these differences were not statistically significant when compared to those receiving non-surgical treatments, such as primary radiotherapy for stage 2 and chemoradiation for stage 4. Among the various treatment methods, hemilaryngectomy and supraglottic horizontal resection provided the longest survival rates in the entire study population.

Previous studies indicate that survival rates in laryngeal cancer vary widely based on the stage of the disease and other potential contributing factors. For example, Cui et al. examined the 5-year survival rates in laryngeal cancer, considering factors such as age, smoking history, ‘*N’*-stage, and various laboratory testing parameters. The 5-year OS rate was 63.5% in the training group and 67.7% in the validation group, according to their findings. Using the aforementioned parameters in a nomogram significantly improved the prediction of survival compared to using only the TNM system [31]. Considering the stages of the disease, a previous study reported a three times higher risk of death in laryngeal cancer at stages 3 and 4. However, a higher chance of survival was observed following surgeries [30]. Another study found significantly improved DSS and OS in advanced stages of laryngeal cancers. Furthermore, stage 3 disease, glottic location, female sex, and married status were found to have a positive influence, while black race and increased age were found to have a negative impact on DSS [32]. According to our study, we have observed better survival rates with surgeries, especially in disease stages 1 and 4. Factors such as tumor location, age, and sex did not significantly impact survival in our group. Another investigation achieving long-term follow-up concluded similar survival rates for all primary treatment modalities (i.e., total laryngectomy, chemoradiation and radiotherapy) for T3 cancers, while better survival rates in terms of total laryngectomy with or without adjuvant radiotherapy for T4 cases [33]. In a study comparing surgeries and radiotherapy for early laryngeal cancers (T1–T2–N0), both treatment options were found to be equally effective. The study included supraglottic horizontal resection, hemilaryngectomy, endoscopic laser surgery, and microlaryngoscopic surgery. However, the study also found that radiotherapy resulted in significantly better voice and speech rehabilitation. This suggests that when considering treatment options, the impact on quality of life and functionality after treatment should be considered [34].

Laryngeal cancer affects men more than women, with recent reports indicating lower survival rates for men [35]. Our study also found a higher number of males diagnosed with laryngeal cancer; however, we did not observe any inherent difference considering sex in OS. Sexton et al. came to a similar conclusion that there was no correlation between male sex and poorer survival compared to females in their study [36].

In our study, we observed a high prevalence of smoking and alcohol use, which are primary risk factors for laryngeal squamous cell cancer. However, we found that these factors do not significantly predict survival outcomes. Among comorbidity characteristics, we identified coronary artery diseases as significant predictors of worse survival outcomes, while respiratory diseases–such as COPD – and T2DM were not found to be significant predictors. Previous research consistently indicates that pre-treatment comorbidities are linked to survival in head and neck cancer, with higher comorbidity rates associated with poorer survival outcomes [37–39]. Mulcahy et al. also showed that both age and comorbidities independently affect prognosis in laryngeal cancer due to their impact on non-cancer-related mortality risk [40].

The reported p16-positivity rate in laryngeal cancers varies widely in the literature, ranging from 1 to 58%. In our study, 24.2% of the examined population was p16-positive. In the study conducted by Chung et al., p16-positivity was found in 7.8% of the examined population [41]. In their report, Dogantemur et al. found p16-positivity in 20% of their cases [42]. Studies examining the relationship between p16-positivity and tumour location have shown varied findings. Xu et al. observed significantly higher p16-positivity in supraglottic cases, whereas other studies have not consistently shown such differences. The study by Dogantemur et al. found a strong link between p16-positivity and tumours located in the supraglottic region [42]. They observed that 55.6% of p16-positive cases were located in the supraglottic area. In our study, 38.9% of cases were p16-positive in the supraglottic region, whereas 15.8% were positive in the glottic region.

The treatment for laryngeal squamous cell cancer is generally similar regardless of where the cancer is located, but the outcomes can vary significantly. The treatment approach and TNM staging can greatly influence survival prognosis. Our study emphasises the continued importance of surgery in treating laryngeal cancer, whether it is in the early or advanced stages. The current treatment options for managing laryngeal cancer include total laryngectomy, hemilaryngectomy, supracricoid horizontal partial laryngectomy, supraglottic horizontal resection, chemotherapy, and radiotherapy. According to our findings, the highest OS was observed in cases where hemilaryngectomy was performed, with a median survival of 156 months, followed by supraglottic horizontal resection at approximately 154 months. Conversely, patients who underwent radiotherapy or chemotherapy had the lowest OS. Molina-Fernández et al. found that patients who underwent surgery had better cause-specific survival and disease-free survival at five years compared to those treated with radiation therapy alone [43]. Sexton et al. found that radiotherapy for supraglottic lesions resulted in worse DSS survival outcomes [36]. In our investigation, comparison of treatment outcomes across ‘*T’* stages showed that supraglottic horizontal resection, supracricoid horizontal partial laryngectomy, and total laryngectomy generally yielded better outcomes compared to other treatment methods studied.

Partial larynx surgeries are essential considering patients’ functionality and quality of life. Following a total laryngectomy, patients require a tracheostomy tube and encounter more challenges in speech rehabilitation. The most commonly reported issues by patients include loss of verbal communication, tracheostoma-related problems such as increased secretion, cough, and frequent upper-airway infections, olfactory problems like hyposmia or anosmia, and emotional and sexual challenges [44]. Following radiotherapy, some late complications can also be observed. These may include xerostomia (permanent loss of saliva), osteoradionecrosis, hypothyroidism, pharyngoesophageal stenosis resulting in dysphagia, lymphoedema, dizziness, and lightheadedness [45]. Hence, partial surgeries should be taken into consideration in order to prevent complications and enhance functionality. A previous study comparing monocentric and multicentric supraglottic cancers treated with partial laryngectomies showed no significant increase in nodal metastasis rates [46]. Another study found that transoral laser surgery yielded similar or better results for supraglottic cancers compared to traditional open supraglottic surgeries or total laryngectomy, regardless of the stage. Furthermore, transoral laser surgeries demonstrated significantly better outcomes than radiotherapy in advanced stages and slightly better results in early stages [47]. In a previous study, supracricoid partial horizontal laryngectomy was found to yield positive oncological outcomes. The study reported 85.8% 3-year OS, 79.1% 5-year OS, 57.6% 10-year OS, and 57.6% 16-year OS rates for patients. Importantly, postoperative radiotherapy was not administered in most cases to improve functional outcomes, with the exception being invasive carcinoma cases [48]. Therefore, partial surgeries should be prioritised when local tumour spreading and the patients’ general health status allow. Furthermore, personalised treatment selection is essential before treating advanced laryngeal cancers, according to the results of a previous investigation, which yielded good outcomes using open partial laryngectomies followed by adjuvant chemoradiation [49].

The strengths of this examination are that it included a large number of patients suffering from laryngeal cancer and achieved long-term follow-up. Furthermore, the study analysed survival rates for different treatment methods, rather than focusing on just one specific treatment option as in previous investigations. This allowed for a comparison of OS rates among different treatment options and disease stages. However, this study had some limitations. First, since the treatment was divided into various groups, the distribution was quite different in each group, which could result in bias when conducting statistical analysis. For instance, the surprisingly low survival rates, considering transoral laser cordectomy - among other possible influencing factors - might be explained by this. Furthermore, some comorbidities that appeared in a low percentage of the population could not be analysed in terms of survival, which could also impact patients’ OS. Furthermore, achieving long-term follow-up can result in significantly different follow-up times for several patients.

## Conclusions

When considering treatment options for laryngeal cancers, this research has shown that the longest survival has been observed after hemilaryngectomy and supraglottic horizontal resection. These procedures not only lead to better survival but also help in preserving organ function. Therefore, partial larynx surgeries should be considered when local tumour spreading allows. Factors influencing survival included the presence of coronary artery disease. Furthermore, poorer survival outcomes are anticipated for cases with higher ‘*N*’ stages, ECOG performance scores, and tumour grades. In considering both surgical and non-surgical treatment options at various stages of the disease, significantly longer survival rates were observed for surgical treatment in stages 1 and 4. Given the study design, future research should investigate factors influencing survival in larynx cancers.
